# Supplementary material for: High-sensitive spatially resolved T cell receptor sequencing with SPTCR-seq
Source: Nat Commun. 2023 Nov 16;14:7432. doi: 10.1038/s41467-023-43201-6 (PMC10654577; doi:10.1038/s41467-023-43201-6)
Supplement: Supplementary file 1 — Supplementary Information [file 41467_2023_43201_MOESM1_ESM.pdf]

Supplementary Information

High-sensitive spatially resolved T cell receptor sequencing with SPTCR-seq

Jasim Kada Benotmane[1,2,3], Jan Kueckelhaus[1,2,3], Paulina Will[1,2,3], Junyi Zhang[1,2,3], Vidhya M. Ravi[1,2,3,4,5], Kevin Joseph[1,2,3,4], Roman Sankowski[8] Jürgen Beck[1,2], Catalina Lee-Chang[7], Oliver Schnell[1,2,3,4], **Dieter Henrik Heiland**[1,2,3,7,8§]

[1]*Department of Neurosurgery, Medical Center - University of Freiburg, Freiburg, Germany*
[2]*Faculty of Medicine, Freiburg University, Germany*
[3]*Microenvironment and Immunology Research Laboratory, Medical Center - University of Freiburg, Freiburg, Germany*
[4]Translational *NeuroOncology Research Group, Medical Center - University of Freiburg, Freiburg, Germany*
[5]*Center for NeuroModulation (NeuroModul), University of Freiburg, Freiburg, Germany*
[6]*Institute of Neuropathology, Medical Center - University of Freiburg, Freiburg, German*
[7]*Department of Neurological Surgery, Northwestern University Feinberg School of Medicine, Chicago, USA*
[8]*German Cancer Consortium (DKTK), partner site Freiburg.*

[§]Lead contact and corresponding author

DISCLOSURE OF CONFLICTS OF INTEREST: No potential conflicts of interest were disclosed by the authors.

Corresponding author:
Dieter Henrik Heiland
Microenvironment and Immunology Research Laboratory
Department of Neurosurgery
Medical Center University of Freiburg
Breisacher Straße 64
79106 Freiburg
-Germany-
Tel: +49 (0) 761 270 50010
Fax: +49 (0) 761 270 51020
E-mail: dieter.henrik.heiland@uniklinik-freiburg.de

Supplementary Figures

Supplementary Figures:

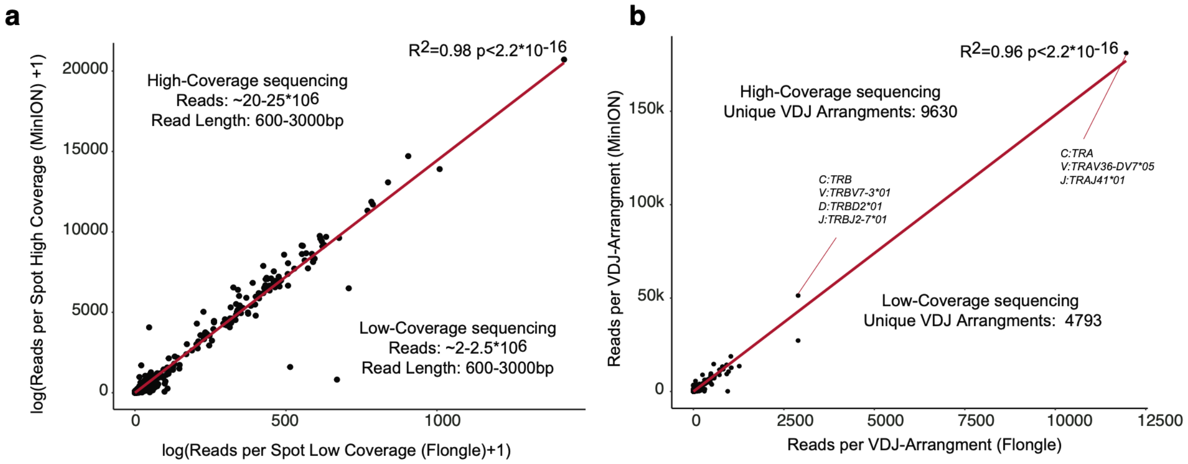

Supplementary Figure 1: a) Correlation plot of reads per spot using both the Flongle (x-axis) and MinION (y-axis) sequencing device. b) Correlation analysis of the number of reads per clone detected by Flongle (x-axis) and MinION (y-axis).

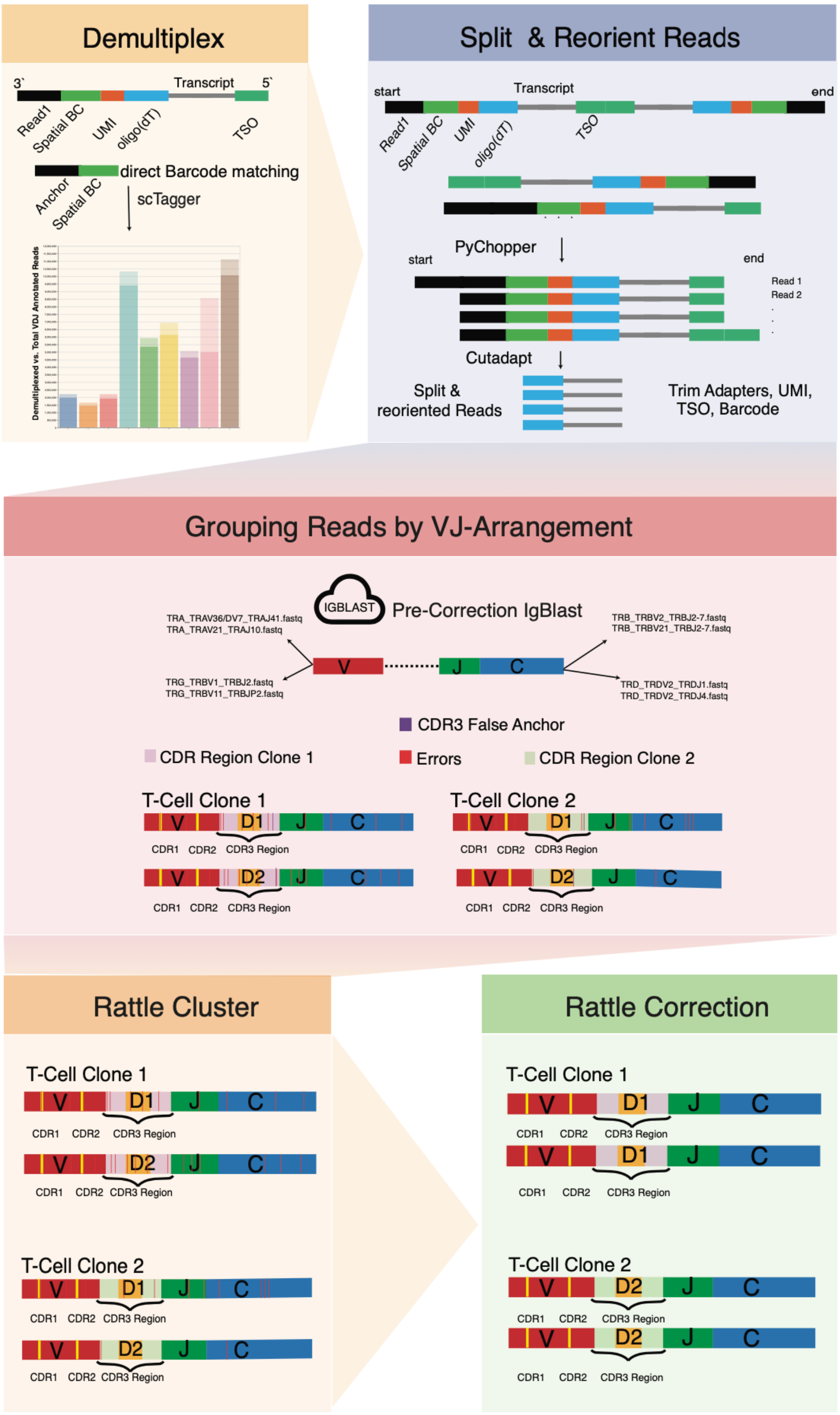

Supplementary Figure 2: Illustration of the SPTCR pipeline.

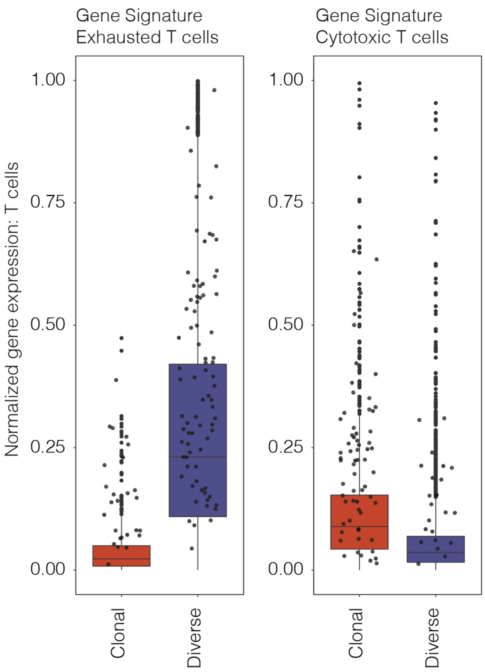

Supplementary Figure 3: a) Enrichment of exhausted and cytotoxic gene sets in regions of clonal or diverse T cell receptor annotations.

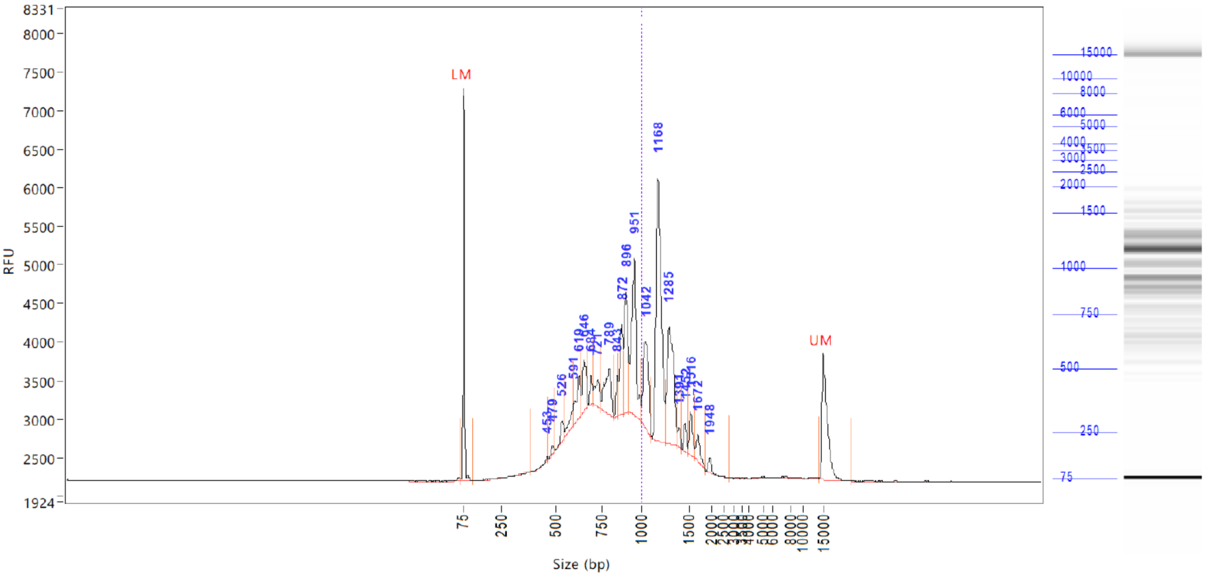

Supplementary Figure 4: Exemplary fragment analyzer traces following SPTCR target enrichment.

Supplementary Note

<u>SPTCR-seq Protocol</u>

1. Experimental Design

For the results described in the SPTCR-seq paper, 11 fresh frozen samples of Glioblastoma multiforme patients were processed using the 10X Visium protocol according to the manufacturer's instructions. **This Protocol was developed based on the KAPA HyperCap Workflow v3.0 October 2020.**

Visium Protocol

Tissue Collection and RNA quality control

Fresh tissue samples were collected immediately after resection and snap-frozen in isopentane after embedding in Tissue-Tek O.C.T. Compound. The embedded tissue was stored at -80°C until further processing. RNA integrity was determined, and samples with an RNA integrity number greater than 7 were used for subsequent analysis.

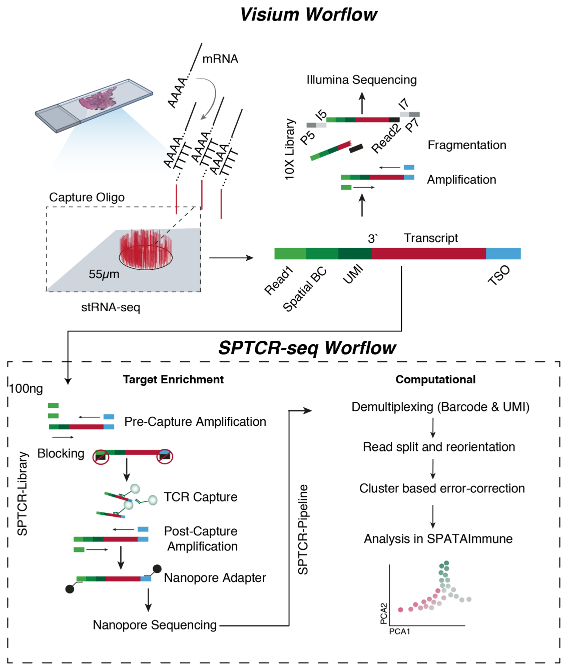

Spatial Gene Expression Protocol

Sections of 10 mm thickness were mounted onto glass slides with poly-T reverse transcription primers, with one section per array and spatial barcoding. Methanol fixation and H&E staining were performed, followed by brightfield imaging at 10x magnification and post-processing with ImageJ software. mRNA from the tissue was captured onto the slide by introducing template switch oligos and performing permeabilization for a predetermined time. Reverse transcription was carried out, and the second strand was cleaved off by denaturation. The resulting cDNA was amplified and fragments of the desired size were selected using SPRIselect reagent. Fragmentation and double-sided size selection were carried out to optimize cDNA fragments for Illumina NextSeq sequencing. Libraries were prepared with unique indexes and Illumina primers and sequenced on the Illumina NextSeq 550 platform using paired-end sequencing. The average length of the final libraries was quantified, and the concentration was determined before dilution and pooling for sequencing. The sequencing was performed using a NextSeq 500/550 High Output Kit v2.5 with 28 cycles for read 1, 10 cycles per index, and 120 cycles for read 2.

SPTCR-seq Protocol

SPTCR-seq Probe Design

In this study, we utilized genomic coordinates of the human genome (hg38/GRCh38) to identify functional T-Cell receptor sequences falling on the TRA, TRB, TRD, and TRG loci from the IMGT database (Giudicelli et al., 2006) to generate probes for target enrichment. The probes were manufactured by Roche and designed using the online tool KAPA Hyperdesign (https://www.hyperdesign.com). To ensure high specificity, a maximum of 20 matches to the human design was allowed, and all probes had less than 6 matches to the human genome. A default value of maximum 100bp of overhang was allowed for window placement of the

genomic segment to ensure comprehensive coverage of the T-cell receptor segments. The design covered a total of 186 regions with 211 unique capture probes, and a list of genes is available in Supplementary Data 2.

Pre-capture Amplification

Prior to target enrichment, the library underwent pre-capture amplification. Specifically, 100 ng of unfragmented cDNA, dissolved in 20 µl of nuclease-free water, was subjected to PCR amplification. A primer mix consisting of 1.5 µl TSO (100 µM), 1.5 µl R1 (100 µM), and 2 µl nuclease-free water was added to the PCR mix. The final volume was brought to 50 µl by adding 25 µl of KAPA HotStart HIFI Ready-mix (Roche Kapa Biosystems) to the reaction. The PCR was carried out under the following conditions: Step 1: 98°C for 3 min, {Step2: 98°C for 20s, Step3: 65°C for 30s, Step4: 72°C for 2 min} x 5 cycles, 72°C for 3 min, 4°C HOLD. Following PCR, transcripts greater than ~150 bp were selected using Roche Kapa HyperPure Beads through a SPRI bead selection procedure, as per the manufacturer's instructions for NGS workflows. Finally, the library was quantified using a Thermofisher Qubit system (1X high-sensitivity dsDNA Kit), and size distribution was measured using an Agilent 5300 Fragment analyzer system operated with the Agilent DNF-920 Reagent Kit to ensure the proper amplification of the library.

Target Enrichment

For this study, we used 1.5µg of the amplified, unmultiplexed cDNA library, which was then filled with PCR-grade water to a final volume of 45µl. To block repetitive regions in the Sample and increase the on-target Rate of the Enrichment, we added COT-Human DNA (included in Roche HyperCapture Reagent Kit) and Blocking Oligos (Partial R1: 5'-CTACACGACGCTCTTCCGATCT-3', Partial TSO: 5'-AAGCAGTGGTATCAACGCAGAG-3', ordered from Thermofisher) to the sample and performed a SPRI-bead cleanup using 130µl of KAPA HyperPure beads. The bound cDNA was then eluted from the beads by pipetting the following agents sequentially directly above the magnet-bound, dried beads: 2.5µl of TSO (100µM), 2.5µl of Read 1 (100µM), and 8.4µl of PCR-grade H2O. The resulting bead suspension (13.4µl) was mixed thoroughly. Next, a hybridization mix comprised of 28µl of Roche hybridization buffer, 12µl of Roche hybridization component H, and 3µl of PCR-grade water was mixed and added to the bead suspension. Finally, 56.4µl of the eluate was mixed with 4µl of the KAPA target enrichment probes that were prepared up to the manufacturer's instructions. Following 5 minutes of double-strand denaturation at 95°C, the solution was incubated for 16 hours at 55°C. The samples were kept at 55°C throughout the remaining steps of the procedure.

Wash and Recover

Prepare the wash buffers according to the manufacturer's protocol for as described in full detail below. Next, prepare the HyperCap Capture Beads according to the manufacturer's protocol for capture target sizes less than 40 Mbp. Mix the sample, after the 16h incubation with the target enrichment probes, with the prepared capture beads and incubate for 15 minutes. Wash the capture beads and elute the bead-bound DNA with the prepared wash buffers of decreasing salt concentration and temperature following the manufacturer's instructions for KAPA HyperCap. Finally, elute the final bead-bound DNA in PCR grade water.

On-Target Amplification

The bead-bound target enriched T-cell receptor transcripts are subjected to a final round of PCR amplification using 2.5µl 10X primers TSO and Read 1 each at 20µM, along with 25µl Kapa HiFi Hotstart ReadyMix and 20µl of the sample. The PCR conditions are as follows: Step 1: 98°C for 3min, {Step2: 98°C for 20s, Step3: 65°C for 15s, Step4: 72°C for 2min} x 18 Cycles, Step5: 72°C for 3min, Step6: 4°C HOLD. The purified product is then subjected to SPRI Bead Cleanup with 70µl KAPA HyperPure Beads according to the manufacturer's instructions. The final purified result is resuspended in 22µl Tris-HCl, of which 2µl can be used for Qubit-quantification and size distribution measurement with the Agilent Fragment analyzer operated with the Agilent DNF-920 Reagent Kit. The remaining sample can be stored at -20°C for several months.

Nanopore Sequencing

To prepare the enriched cDNA for sequencing, we employed Oxford Nanopore Technologies (ONT) 1D sequencing. For the Samples #UKF260GBM, #UKF334GBM, #UKF248GBM, #UKF275GBM, #UKF304Recurrent, #UKF269GBM, #UKF313GBM, #UKF296GBM, we utilized the sequencing by ligation sequencing kit (SQK-LSK110) and the FLO-MIN106D with R9.4 Chemistry. For the UKF454Primary and UKF454 Recurrent samples, we employed FLO-MIN114 with the R10.4.1 Flow cells and the Library Preparation for Sequencing by Ligation V14. The library preparation workflow for the Samples #UKF260GBM, #UKF334GBM, #UKF248GBM, #UKF275GBM, #UKF304Recurrent, #UKF269GBM, #UKF313GBM, #UKF296GBM, was performed up to the manufacturer's protocol (GDE_9108_v110_revE_10Nov2020.pdf), using an input of 200-300fmol. We opted to enrich for fragments <3kb following adapter ligation, utilizing the provided Short Fragment Buffer. For the final loading step, ~5FMOL of the sequencing library was utilized, and samples were sequenced individually on single flow cells.

As ONT sequencing is a rapidly evolving third-generation sequencing technique that is constantly improving, we do not describe library preparation techniques. Rather it is recommended that users select the most recent and established sequencing by ligation kit, as well as the most current and established base calling method to achieve optimal results.

With the rapid evolution of ONT sequencing, users should to remain up-to-date with the latest advancements to ensure the most accurate and reliable sequencing results. Listed Kits were used for publication.

2. Materials

Equipment

Name	***Company***
PCR-Strip, 0,2 ml	Starlab
QubitTM Assay Tubes	Thermo Fisher Scientific Inc.
Agilent Fragment Analyzer	Agilent Technologies Inc.
MinION Sequencer	Oxford Nanopore Technologies
Qubit 4 Fluorometer	Thermo Fisher Scientific Inc.
Magnet Stand for PCR Strips	

Microfuge for PCR Strips	
Vortex mixer	
Thermocycler with programmable heated lid (Capable of maintaining +55°C for 16 - 20 hours)	

Reagents

Target Enrichment

Name	*Company*	*ProductID*	*Description*
KAPA HyperCapture Bead Kit	Roche	9075780001	Target capture biotinylated beads
KAPA HyperCapture Reagent Kit	Roche	9075810001	Capture Reagent Kit for Target Enrichment
KAPA HyperPure Beads	Roche	08963835001	SPRI Beads for Size Selection and Cleanup
KAPA Probes Resuspension Buffer	Roche	9075879001	Resuspension Buffer for KAPA HyperChoice MAX 0.5Mb
KAPA HyperChoice MAX 0.5Mb	Roche	9052356001	Custom designed capture oligos,<br>IRN: 0200533939
**Custom Adapter Oligos*	ThermoFisher		Order from Thermofisher HPLC purified:<br>**Partial R1**: 5'-CTACACGACGCTCTTCCGATCT-3'<br>**Partial TSO**: 5'-AAGCAGTGGTATCAACGCAGAG-3'
KAPA HiFi HotStart ReadyMix	Roche	7958927001	PCR Ready Mix for Amplification

* Adapter oligos can serve dual purposes of amplifying and blocking flanking adapters during target enrichment. If used as blocking oligos during target enrichment, full length HPLC Purification is recommended. The oligos can be interchanged to adapt the workflow as needed.

Oxford Nanopore Sequencing

Name	*Company*	*ProductID*	*Type*
Ligation Sequencing Kit (SQK-LSK110)	Oxford Nanopore Technologies	SQK-LSK110	Library Preparation Kit V10 for ONT Sequencing
Ligation Sequencing Kit V14 (SQK-LSK114)	Oxford Nanopore Technologies	SQK-LSK114	Library Preparation Kit V14 for ONT Sequencing
NEBNext® Companion Module for Oxford Nanopore Technologies® Ligation Sequencing	NEB Bio	E7180S	Companion Module needed for ONT Library Preparation Kit

3. Preparation

1. **Resuspend the Target Enrichment Probes** following arrival in the Probe Resuspension Buffer according to the Manufacturers Protocol
2. **Aliquot the Probes into 4µl Aliquots.**
3. **Resuspend the Partial Read 1 and Template Switch Oligo Primers ordered to 100µM.**
    a. Dilute to 3µM for Pre-Capture PCR
    b. Dilute to 20µM for Post Capture PCR
4. **Prepare 100ng of input cDNA** from the Visium Protocol Step 3.3 (Visium Spatial Gene Expression Reagent Kits, CG000239 Rev C)

4. Procedure

Pre-Capture Amplification ~1h

Needed Reagents:

- KAPA HyperPure Beads
- Prepared 10X partial Read 1 100µM and Partial Template Switch Oligo (partial TSO)
- PCR Grade Water
- RNAse free water
- RNAse free Ethanol

1. **Dissolve 100ng of Visium cDNA** in **20 µl** of PCR Grade Water.
2. Add **2 µl** of PCR Grade Water.
3. Add **1.5 µl of each TSO** and **Partial Read 1 100µM Primer**.
4. Add **25µl KAPA HiFi HotStart ReadyMix** to each Sample.
5. Mix the samples thoroughly and perform a quick spin. Immediately proceed to amplification.
6. Place the Samples in the Thermocycler, and perform the following program with the lid temperature set to +105°C.
    a. **Step 1: 98°C 3min**
        i. **98°C for 20**
        ii. **65°C for 30s**
        iii. **72°C for 2min**
            ⇨ **5Cycles**
    b. **Step 3: 72°C 1min**
    c. **HOLD**
7. Immediately after the amplification perform an **SPRI Bead Cleanup with KAPA HyperPure Beads**
    1. Add **1.4X (70 µL) of KAPA HyperPure Beads** at room temperature to each amplified sample library, ensuring that the beads are thoroughly resuspended.

    2. Thoroughly mix the amplified sample library and KAPA HyperPure Beads, followed by a quick spin.
    3. Allow the DNA to bind to the beads by incubating the sample at room temperature for **5 minutes**.
    4. To collect the beads, place the sample on a magnet and incubate it until the liquid becomes clear.
    5. Carefully remove and discard the supernatant.
    6. While keeping the sample on the magnet, add **200 µL of 80% ethanol**, which should be freshly prepared.
    7. Incubate the sample at room temperature for at least 30 seconds.
    8. Carefully remove and discard the ethanol, being careful not to disturb the beads.
    9. **Repeat Step 6-8**, resulting in two washing steps.
    10. Allow the beads to dry at room temperature until all the ethanol has evaporated.
    11. Remove the sample from the magnet.
    12. Resuspend the beads using 30µl of **10mM Tris-HCL, pH 8.0**
    13. Incubate at room temperature for **2minutes** to elute the DNA off the beads.
    14. **Transfer 30µl of Eluate** to a new tube/well
    15. The amplified library is now read for target capture. It can be stored at +2°C to +8°C for 1-2 weeks or for long term storage frozen (-15°C to -20°C)
2. **Measure the Concentration and Size Distribution** of the Amplified Sample Library using the Qubit Fluorometer and the Agilent Fragment Analyzer

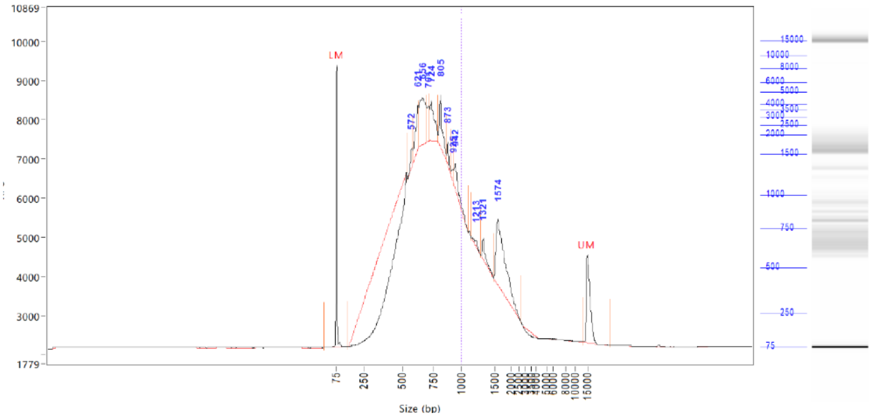

Figure 1 Exemplary Size Distribution of amplified pre-capture 10X Visium Library

The Samples should contain **1000ng of correctly amplified Library** for the Target Enrichment.

Probe Binding ~1h

Needed Reagents:

- KAPA HyperPure Beads
- KAPA HyperCapture Reagent Kit
    - COT Human DNA
    - Hybridization Component H
    - Hybridization Buffer
- Prepared 10X partial Read 1 100µM and Partial Template Switch Oligo (partial TSO)
- PCR Grade Water
- RNAse free Ethanol

1. **Thaw the appropriate number of 4 µL KAPA Target Enrichment Probe aliquots** (one per hybridization) from the -15 to -25°C freezer on ice.
2. **Prepare Blocking Oligos for the 10X Sequencing Adapters**

REAGENT	1X	+10%
**PARTIAL TEMPLATE SWITCH OLIGO (PARTIAL TSO) 100µM**	2.5µl	2.75µl
**PARTIAL READ 1 (R1) 100µM**	2.5µl	2.75µl
**PCR GRADE WATER**	8.4µl	9,24µl

3. If capturing a single sample, utilize **1000 ng of the amplified Visium** library.
4. Add PCR Grade water to achieve a **final volume of 45 µL**.
5. To the DNA Sample Library Pool, add **20 µL of COT Human DNA** (1 mg/mL, contained in KAPA HyperCapture Reagent Kit) resulting in a total volume of 65 µL.
6. **Add 130 µL of KAPA HyperPure Beads**.
7. **Vortex** for 10 seconds and perform a quick spin to mix the contents.
8. **Incubate** the sample at room temperature for **10 minutes** to allow the DNA Sample Library and COT Human DNA to bind to the beads.
9. Place the sample on a **magnet** to collect the beads. Incubate until the liquid is clear.
10. Carefully remove and **discard the supernatant**.
11. Keeping the sample on the magnet, **add 200 µL of freshly-prepared 80% ethanol**.
12. Incubate the sample at room temperature for at least 30 seconds.
13. Carefully remove and discard the ethanol, taking care to remove residual ethanol without disturbing the beads.
14. Allow the beads to dry at room temperature until all the ethanol has evaporated.
15. **Add 13.4 µL of Blocking** Oligos to the bead-bound DNA sample. Pipet directly on the bead bound Sample.
    - ⇨ Aim 8.3µM Concentration Blocking Oligos in the **60.4µl** Target Enrichment Reactions
16. Remove the sample from the magnet and vortex thoroughly to ensure a homogenous mixture.
17. Prepare the **Hybridization Master Mix** following the tables below according to the capture target size.

Reagent	+10%
**Hybridization Buffer**	30.8
**Hybridization Component H**	13.2
**PCR Grade water**	3.3
**Total**	**47.3**

18. Add **43 µL of the Hybridization Master Mix** to the bead-bound DNA mixture resuspended in Universal Enhancing Oligos.
19. Mix thoroughly and perform a quick spin. Incubate at room temperature for 2 minutes.
20. Place the sample on the magnet to collect the beads. Incubate until the liquid is clear.
21. **Transfer 56.4 µL of the eluate (entire volume)** into a new tube/well containing 4 µL of the KAPA **Target Enrichment Probe**.
22. Vortex thoroughly for 10 seconds and perform a quick spin to mix the contents.
23. **Incubate the sample in a thermocycler** using the following program with the lid temperature set to +105°C:
    1. **+95°C for 5 minutes**
    2. **+55°C for 16 hours**

Capture Bead Binding and Washing ~2h

Needed Reagents:

- KAPA HyperPure Beads
- KAPA HyperCapture Bead Kit
- KAPA HyperCapture Reagent Kit
- Prepared 10X partial Read 1 100µM and Partial Template Switch Oligo (partial TSO)
- PCR Grade Water
- RNAse free water
- RNAse free Ethanol

Preparation ~30min

Wash Buffers

CONCENTRATED BUFFER	VOLUME OF CONCENTRATED BUFFER	VOLUME OF PCR GRADE WATER	TOTAL VOLUME OF 1X BUFFER	TEMPERATURE
**10X STRINGENT WASH BUFFER**	44 µL	396 µL	440 µL	+55°C
**10X WASH BUFFER I**	11 µL	99 µL	110 µL	+55°C
	22 µL	198 µL	220 µL	Room temp.
**10X WASH BUFFER II**	22 µL	198 µL	220 µL	Room temp.
**10X WASH BUFFER III**	22 µL	198 µL	220 µL	Room temp.
**2.5X BEAD WASH BUFFER**	132 µL (2x66µl)	198 µL (2x99µl)	330 µL	Room temp.

1. Before the completion of the hybridization incubation, ensure that the Hybridization Wash Buffers are thawed. **It is important to note that the stock wash buffers should not be precipitated or cloudy**. If necessary, vortex the cloudy buffers thoroughly and warm them at 37°C until they are completely clear.
2. Dilute the 10X Wash Buffers (I, II, III, and Stringent) and the 2.5X Bead Wash Buffer, which are included in the KAPA HyperCapture Reagent Kit, to **create 1X working solutions**. The volumes specified are sufficient for a single capture and include +10% Excess.
3. To pre-warm the **1X Stringent Wash Buffer, two aliquots of 220 µL each** are made in 0.2 mL tubes, and these tubes are placed into a thermocycler set to **+55°C**.
4. To pre-warm the 1X Wash Buffer I, one aliquot of 100 µL is made in a 0.2 mL tube and placed into a thermocycler set to +55°C.
5. **Buffers are pre-warmed for a minimum of 15 minutes**.
6. The pre-warming of buffers can be performed in the same thermocycler used in the probe hybridization incubation step.

Capture Beads

1. Allow the **Capture Beads to equilibrate to room temperature** prior to use
2. **Vortex** the Capture Beads for 15 seconds to mix them well.

3. Transfer **50 µL of beads** per capture reaction into a tube. Use a 0.2 mL or 1.5 mL tube depending on the number of captures (e.g. 200 µL beads for four captures in a single 0.2 mL tube or up to twelve captures in a single 1.5 mL tube).
4. **Put the Beads on a Tube holding magnet** to collect the beads and wait until the liquid becomes clear.
5. Remove the liquid carefully without disturbing the beads.
6. **Add 2X the initial volume of 1X Bead Wash Buffer** to the beads while keeping the tubes on the magnet (e.g. 100 µL of buffer for one capture or 400 µL for four captures).
7. Take the tubes off the magnet and shake them well. Then perform a quick spin.
8. Put the tubes on the magnet and wait until the liquid is clear.
9. Remove the liquid without disturbing the beads.
10. **Perform a second wash by adding 2X the initial volume of 1X Bead Wash Buffer while keeping the tubes on the magnet** (e.g. 100 µL of buffer for one capture or 400 µL for four captures).
11. **Repeat Steps 7-9**.
12. **Add 1X the initial volume of 1X Bead Wash Buffer** (i.e. 50 µL buffer per capture).
13. Take the tubes off the magnet and vortex them well for 10 seconds. Then perform a quick spin.
14. **Transfer 50 µL of resuspended beads into a <u>new tube/well</u>** for each capture.
15. Put the tubes on a magnet and wait until the liquid is clear.
16. **Remove the liquid carefully**.
17. The Capture Beads are now ready to bind the hybridized DNA. Proceed to the next step immediately and **do not allow the Capture Beads to dry out**. Small amounts of residual Bead Wash Buffer will not affect DNA binding to the Capture Beads.

Bead Binding ~20min

1. **Transfer each hybridization sample into a single tube or well containing the prepared Capture Beads** from the previous step.
2. Mix the sample and beads thoroughly by vortexing for 10 seconds, followed by a quick spin.
3. **Incubate the hybridization reaction by placing the sample in a thermocycler set to +55°C** for **15 minutes**, with the thermocycler lid temperature set to +105°C. Bead binding can be performed in the same thermocycler used in the probe hybridization incubation step.
4. Proceed immediately to the next step without delay.

Wash Bead Bound DNA ~1h

1. To the Hybridization reaction, add **100 µL of pre-warmed 1X Wash Buffer I.**
2. Vortex for 10 seconds to ensure homogeneity and perform a quick spin.
3. Place the sample on a magnet and let it incubate until the liquid becomes clear.
4. Carefully remove the supernatant without disturbing the beads.
5. Add **200 µL of pre-warmed 1X Stringent Wash Buffer** to each sample.
6. Remove the sample from the magnet and vortex for 10 seconds to ensure homogeneity. Perform a quick spin.
7. Place the sample in the thermocycler preheated to +55°C, close the lid (set to +105°C) and ***incubate for 5 minutes***.
8. Remove the sample from the thermocycler after the 5 minutes incubation and place it on a magnet to collect the beads.
9. Incubate until the liquid is clear and carefully remove the supernatant without disturbing the beads.
10. **Repeat Steps 5-9 using 200 µL of pre-warmed 1X Stringent Wash Buffer**
11. Add **200 µL of 1X Wash Buffer I at room temperature**.
12. Vortex for 10 seconds to ensure homogeneity and perform a quick spin.
13. Let the sample ***incubate at room temperature for 1 minute***.
14. Place the sample on a magnet and incubate until the liquid is clear.
15. Carefully remove the supernatant without disturbing the beads.
16. Add **200 µL of 1X Wash Buffer II at room temperature**.
17. Vortex for 10 seconds to ensure homogeneity and perform a quick spin.
18. **Transfer Contents to a new Tube**
19. Let the sample ***incubate at room temperature for 1 minute***.
20. Place the sample on a magnet and incubate until the liquid is clear.
21. Carefully remove the supernatant without disturbing the beads.
22. **Repeat Steps 11-15 using 200 µL of room temperature 1X Wash Buffer III**.
23. Remove the sample from the magnet and add **20 µL of PCR Grade water** to each tube/plate well. Vortex for homogeneity and perform a quick spin.
24. **Proceed to Amplification of Enriched Multiplex DNA Sample**. No elution step is performed at this step. The beads contain the captured DNA libraries and will be used as the template in the PCR.

Target Amplification ~1h

Needed Reagents:

- KAPA HyperPure Beads
- KAPA HiFi HotSTART ReadyMix
- Prepared 10X partial Read 1 100µM and Partial Template Switch Oligo (partial TSO)
- PCR Grade Water
- RNAse free Ethanol

1. **Prepare a diluted aliquot of 20µM 10X Primers Read1 and TSO** by adding 1µl of 100µM Primer to 4µl PCR grade water.
2. Bring **KAPA HyperPure Beads to Room Temperature**
3. Prepare the **Post Capture PCR Master Mix**

REAGENT	FOR ONE REACTION +10%
**KAPA HIFI HOTSTART READYMIX (2X)**	27.5µl
**TSO 20µM**	2.75µl
**R1 20µM**	2.75µl

4. Using a fresh 0.2 mL tube or well of a PCR plate and add **<u>30 µL of Post-Capture PCR Master Mix</u>** to it.
5. Retrieve the **bead-bound DNA from Chapter 6 and mix it thoroughly by vortexing** for 10 seconds. Perform a quick spin.
   - ⇨ The total volume of the sample with beads will be around 20 µL (15 µL of water with bead volume). If the volume is less than 20 µL, add more water to achieve this volume.
6. **Transfer 30 µL of Post-Capture PCR Master Mix to a tube/well containing 20 µL of the bead-bound DNA as a template.**
7. **Mix** the contents thoroughly by pipetting up and down several times.
8. Place the Samples in the Thermocycler, and perform the following program with the lid temperature set to +105°C.
   a. **Step 1: 98°C 3min**
      i. **98°C for 20s**
      ii. **65°C for 15s**
      iii. **72°C for 2min**
         - ⇨ **18Cycles**
   b. **Step 3: 72°C 1min**
   c. **HOLD**
      - ⇨ Immediately proceed to SPRI Bead Cleanup
9. Immediately after the amplification perform an **SPRI Bead Cleanup with KAPA HyperPure Beads**
10. Ensure KAPA HyperPure Beads are well equilibrated to Room Temperature and a homogenous mixture.
11. **Prepare 500µl 80% Rnase free ethanol** by mixing 400µl Rnase free ethanol with 100µl Rnase free Water.
12. Place the amplified enriched Multiplex DNA Sample on a magnet to **collect the Capture beads** and **<u>transfer the supernatant to a new tube</u>**.
13.
14. Add **1.4X (70 µL) of KAPA HyperPure Beads** at room temperature to the 50µl target enriched sample library, ensuring that the beads are thoroughly resuspended.

15. Thoroughly **mix** the amplified sample library and KAPA HyperPure Beads, followed by a quick spin.
16. Allow the DNA to bind to the beads by incubating the sample at room temperature for **5 minutes**.
17. To collect the beads, place the sample on a magnet and incubate it until the liquid becomes clear.
18. Carefully remove and discard the supernatant.
19. While keeping the sample on the magnet, add **200 µL of 80% ethanol**, which should be freshly prepared.
20. **Incubate** the sample at room temperature for at least 30 seconds.
21. Carefully remove and discard the ethanol, being careful not to disturb the beads.
22. **Repeat Step 6-8**, resulting in two washing steps.
23. Allow the beads to dry at room temperature until all the ethanol has evaporated.
24. Remove the sample from the magnet.
25. **Resuspend the beads using 22µl** of **10mM Tris-HCL, pH 8.0**
26. Incubate at room temperature for **2minutes** to elute the DNA off the beads.
27. **Transfer 20µl of Eluate** to a new tube/well
28. The target enriched library is now ready to be prepared for sequencing. It can be stored at +2°C to +8°C for 1-2 weeks or for long term storage frozen (-15°C to -20°C)
29. Transfer **20 µL** of the eluate to a new tube/well. This will be our final TCR enriched cDNA, able to be prepared for sequencing.
30. **Dilute 2µl 1:10 with PCR Grade water for Qubit Quantification and Measurement of Size Distribution**.

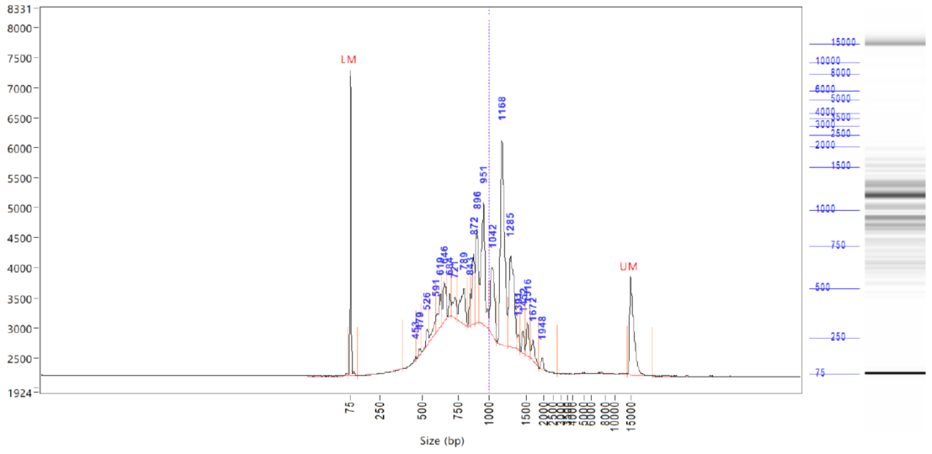

Figure 2 Exemplary Fragment Analyzer Trace following Target Enrichment of a GBM Patients Visium Library

5. Data Analysis

6. Troubleshooting

For Troubleshooting also refer to KAPA HyperCap Workflow. Common Pitfalls encountered are listed below.

1. Input cDNA less than 100ng
    a. Libraries generated with <100 ng of input gDNA can yield high-quality capture results.
    b. Increasing the number of PCR cycles during Pre-Capture PCR by 1-3 cycles can improve results, depending on the starting gDNA amount.
    c. Fill the volume for the target enrichment process to **45 µL** for targeted capture.

2. Pre-capture Sample library volume exceeds 45µl.
    a. add 20 µL of COT DNA to the Multiplex DNA Sample Library in a 1.5 mL tube, followed by 2X the Volume of KAPA HyperPure Beads (adapt to sample Volume, instead of using fixed 130µl). Continue the Protocol as is.
    b. If using a vacuum concentrator, concentrate the sample after adding COT DNA

3. After Cleanup Primer Peak in Fragment Analyzer
    a. If done correctly, might indicate a decay of HyperPure Beads. Replace Beads.
    b. Aliquot in 500µl Tubes to improve longevity of beads.
    c. Might not interfere with target enrichment but will lead to overestimation of input for enrichment.

4. Post Capture PCR lass than 100ng
    a. Pre-Capture PCR yield should be ≥ 1 µg. If lower, repeat with a DNA sample that was previously processed with success.
    b. Verify correct hybridization and wash temperatures were used. If incorrect, repeat experiment from hybridization.
    c. Check PCR reagents for compromise. Verify positive control worked. If not, repeat hybridization and re-amplify using fresh PCR reagents.
    d. Depends also on quality of the Visium Library.

5. Applicability for Visium FFPE
    a. The Workflow was not tested with Visium FFPE and might in doubt not be feasible as the RNA will be too degraded and full-length transcripts might not/only in low abundance be detectable.
    b. Workflow should be done with fresh frozen Samples.
    c. Illumina sequencing and Computation TCR Reconstruction (e.g., MixCR, TRUST4) might possibly be feasible options

6. Adaption to other Sequencing Platforms (e.g., Slide Seq)
    a. The Workflow is easily adaptable for the needs of other protocols as well from fresh frozen Tissue.
    b. Only Adaption needed would be to replace the oligos/primers required to amplify and block the sequencing adapters (for 10X: Read 1, TSO) with platform specific primers. Order them HPLC purified to ensure full length for their blocking functionality.
