# Supplementary material for: High-sensitive spatially resolved T cell receptor sequencing with SPTCR-seq
Source: Nat Commun. 2023 Nov 16;14:7432. doi: 10.1038/s41467-023-43201-6 (PMC10654577; doi:10.1038/s41467-023-43201-6)
Supplement: Supplementary file 3 — Description of Additional Supplementary Files Document [file 41467_2023_43201_MOESM3_ESM.pdf]

Description of Additional Supplementary Files

Supplementary Data:

Supplementary Data 1: Patient Cohort and Assay Information

This table provides a description of the patient cohort and enumerates the individual assays conducted per patient within the study. It offers a methodical organization of participant-specific data and corresponding experimental procedures.

Supplementary Data 2: Laboratory Equipment and Reagents

This table catalogs the laboratory tools and reagents employed for the realization of the protocol and execution of the experiments. It details T Cell Receptor (TCR) HGNC gene symbols, spatial barcodes associated with the Visium protocol, and chromosomal regions targeted for the synthesis of the TCR target enrichment probes.
